# Fatty Infiltration of the Myocardium and Arrhythmogenesis: *Potential Cellular and Molecular Mechanisms*

**DOI:** 10.3389/fphys.2018.00002

**Published:** 2018-01-22

**Authors:** Justus M. B. Anumonwo, Todd Herron

**Affiliations:** Department of Internal Medicine (Cardiovascular Medicine), Center for Arrythmia Research, University of Michigan, Ann Arbor, MI, United States

**Keywords:** fatty infiltration, myocardial remodeling, obesity, cardiac arrhythmias, epicardial adiposity

## Abstract

Anatomical evidence in several species shows highly heterogeneous fat distribution in the atrial and ventricular myocardium. Atrial appendages have fat deposits, and more so on the posterior left atrium. Although such fat distributions are considered normal, fatty infiltration is regarded arrhythmogenic, and various cardiac pathophysiological conditions show excess myocardial fat deposits, especially in the epicardium. Hypotheses have been presented for the physiological and pathophysiological roles of epicardial fat, however this issue is poorly understood. Therefore, this mini-review will focus on epicardial fat distribution and the (patho)-physiological implications of this distribution. Potential molecular mechanisms that may drive structural and electrical myocardial remodeling attendant to fatty infiltration of the heart are also reviewed.

## Myocardial adipose tissue

Approximately 80% of the myocardial surface is covered by fat, and it is estimated that ~20% of the total heart weight is due to the fat content (Iacobellis et al., 2005; Sacks and Fain, 2007). The distribution of fat on the myocardial surface is species dependent, with more deposits of fat present on large mammals, including humans, compared to small laboratory animals such as rodents (Marchington et al., 1989; Iacobellis et al., 2005). It is surprising that for decades, there remained limited studies in humans regarding the relationship of normal cardiac anatomy and pathology (Iacobellis et al., 2005). Anatomical evidence in humans, as well as in other species, indicates a highly heterogeneous epicardial fat distribution throughout the atrial parenchyma, with increased presence at the appendages, and more so at the posterior left atrium. Fat deposits abound on the free wall of the right ventricle, and the apex of the left ventricle has some amount of fat deposits. Human autopsy reports show that despite the fact that left ventricular mass far exceeds that of the right ventricle, total amount of fat tissue was similar in both ventricles, (Corradi et al., 2004; Iacobellis et al., 2005). Nevertheless, it must be noted that although this epicardial fat distribution is considered normal, fatty infiltration of the myocardium is regarded arrhythmogenic (adiposis cardiaca), and is frequently observed under pathophysiological conditions, as discussed in a later section. Our current thinking is that epicardial fat thickness that is >5 mm, or volume of the tissue greater that 125 mL should be considered abnormal (Bertaso et al., 2013). Several hypotheses have been presented for the physiological and pathophysiological roles of myocardial fat tissue. Adipose tissue found around blood vessels has been suggested to reduce forces (vascular tension and torsion), thus playing a supportive role (Iozzo, 2011). The fatty tissue may also be involved in release of a variety of circulating biofactors, including cytokines and hormones, which are important in modulating myocardial vasculature (Iacobellis et al., 2005; Sacks and Fain, 2007; Iozzo, 2011). Our discussions in this review will emphasize fatty infiltration of myocardium and the (patho)-physiological implications of such a process.

**Figure 1:**
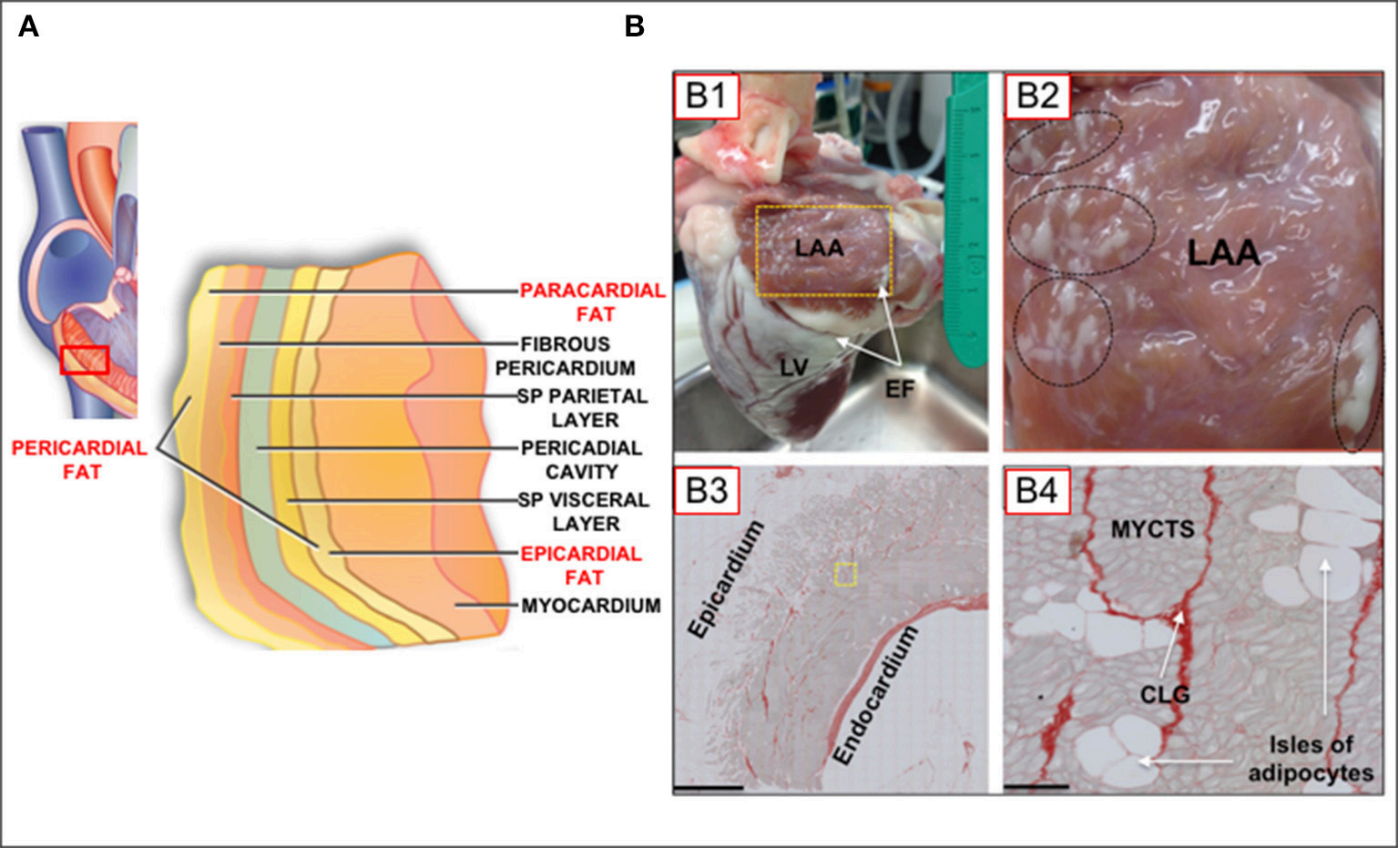
**(A)** Schematic of myocardial fat depots in the myocardial wall of the right ventricle (Modified from Wong et al., 2016). **(B)** Epicardial fat tissue distribution in the ovine heart. **(B)** Normal adult sheep heart showing atrial and ventricular epicardial fat deposits. LAA, left atrial appendage; LV, left ventricle; EF, epicardial fat, on atrial and ventricular surfaces. Yellow box in **(B1)** is 2 × 3 cm. **(B2)**, [Inset of box from **(B1)**] Oval and circles represent regions of dense atrial epicardial fat. **(B3)**, LAA tissue section. Note significant epicardial fat layer with extensive adipocyte infiltration of the atrial muscle (Scale bar; 1mm). **(B4)**, Tissue section of a region near yellow inset in **(B3)**, showing myocytes (MYCTS), isles of adipocytes, and collagen (CLG; Picoserius staining). Scale bar: 50 μm. (Modified from O'Connell et al., 2015).

A variety of imaging technologies have been used to investigate adipose tissue depots on the heart, including echocardiography, computer tomography (CT), and cardiac magnetic resonance imaging (CMRI). Each imaging modality has advantages and limitations (Wong et al., 2016), all of which impact on the choice of a modality for investigative purposes. Thus, for example, whereas complex modalities such as CT and CMRI have the advantage of volume, area, and thickness measurements, echocardiography is widely available and relatively inexpensive (Wong et al., 2016). However, echocardiographic measurements are limited to measurements of thickness. On the other hand, CT scans have the complication of ionization radiation, and CMRI are expensive and may not be technically feasible for extremely obese patients (Wong et al., 2016). Arguably, some of these techniques are less suited for imaging cellular details of fatty infiltrations in the myocardium. In several reports describing myocardial fat layers, various, and sometimes conflicting/confusing terminologies have been used to describe myocardial fat depots (Sacks and Fain, 2007; Thanassoulis et al., 2010; Samanta et al., 2016; Wong et al., 2016). Figure 1A shows different layers of fat tissue on the myocardium, as recently schematized in the review by Wong et al. (2016). As in this schematic, our review will adopt the terminology that denotes pericardial fat as a combination of paracardial and epicardial fat depots. It is noteworthy that both epicardial and paracardial fat depots derive from brown adipose tissue, but from an embryological standpoint (Wong et al., 2016), paracardial fat evolves from the primitive thoracic mesenchyme (Iacobellis et al., 2005), whereas epicardial derives from the gut-related, splanchnopleuric mesoderm (Sacks and Fain, 2007).

Histological analysis of tissue sectioning has provided important information on fat infiltration of the myocardium. Thus, it is known that there are extensive fat deposits on the surface of atria and ventricles (more so for the latter; Figures 1B,B1). However, as shown in this Figure 1B, significant fat deposits are present within the atrial myocardium (Figures 1B,B2), and adipocytes are contiguous with myocytes, with no evidence of a fascia separating each tissue type, in contrast to what is observed in skeletal muscles (Iacobellis et al., 2005; Hatem and Sanders, 2014). Adipocytes express ion channels, including gap junctional channels (Burke et al., 2014), however there is little or nothing known about on adipocyte/myocyte electrical coupling. As pertinent to the discussions in this review, this contiguity between adipocytes and myocytes, as well as the physical proximity between adipocytes/myocytes with other cell-types, readily allows for modulatory effects of adipocyte biofactors on myocytes. Such a scenario is expected to have profound implications for myocardial function and pathophysiological cellular remodeling.

## Characteristics of adipose tissue depots in myocardial diseases

**Figure 2:**
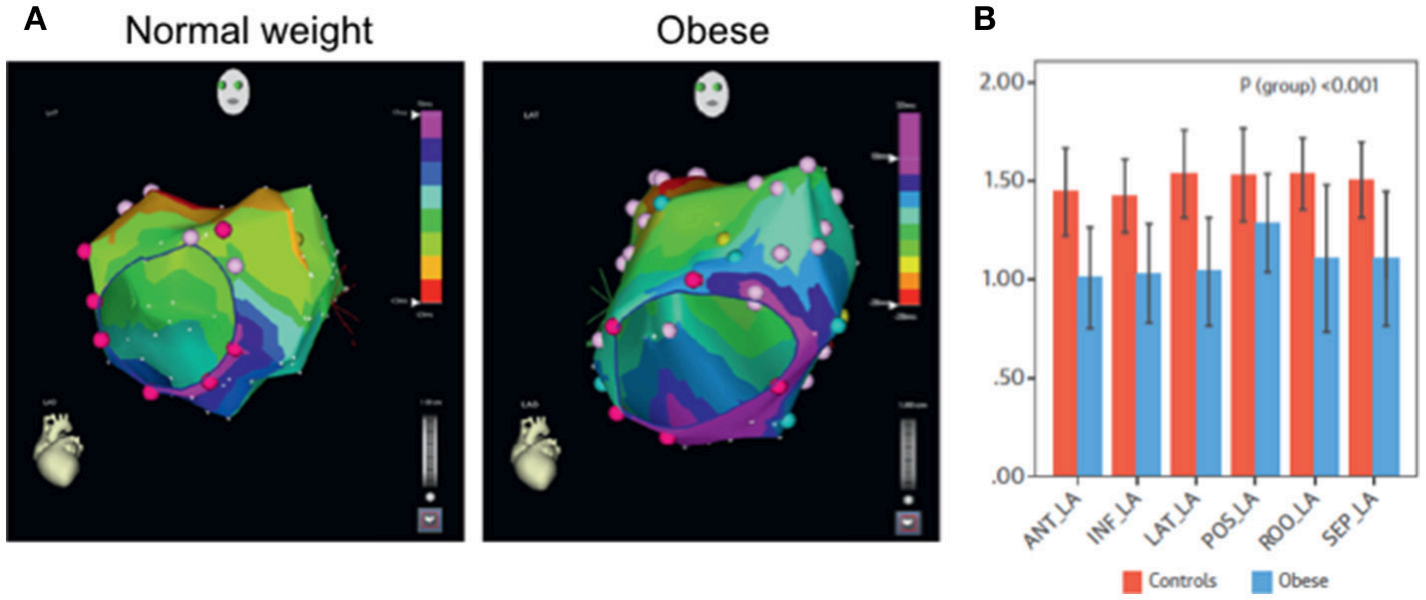
Analyses of Impulse conduction in normal weight and in obese ovine (Measurements during sinus rhythm and isochrones from left anterior oblique view). **(A)** Representative 5 ms isochronal maps. Note crowding of isochrones (i.e., slower propagation time) in the obese ovine, compared to the control. **(B)** Mean values of conduction velocity measured in the specified regions of the left atrium. Note that conduction velocity was reduced uniformly in all regions of the atrial myocardium. ANT, anterior; LA, left atrium; INF_LA, inferior left atrium; LAT_LA, lateral left atrium; POS_LA, posterior left atrium; ROO_LA, left atrial roof; SEP_LA, septal left atrium. Modified from Mahajan et al. (2015).

Epicardial adipose tissue (EAT) is increased in myocardial diseases, an increase that is associated with arrhythmogenicity (Sacks and Fain, 2007; Christensen et al., 2008; Sen-Chowdhry et al., 2010; Deshpande et al., 2016; Samanta et al., 2016). With increase in EAT, it has been suggested excess adiposity, cytokines, free fatty acids and other bioactive molecules are accumulated within the vicinity of myocytes, which are consequently challenged structurally and electrically (Samanta et al., 2016). Such remodeling processes alter normal impulse initiation and propagation properties of the myocardium. In a recent study (see Figure 2), the ovine model was used to determine structural and electrophysiological substrates of atrial fibrillation (AF) (Mahajan et al., 2015). The study showed conduction abnormalities, which was related to infiltration of posterior left atrial muscle by epicardial fat. It was concluded that obesity-induced increase in EAT resulted in structural and electrical remodeling, leading to increased propensity for AF. In general, it is recognized that EAT may act by one or more of the hypothesized arrhythmia mechanisms, involving automaticity, triggered activity or reentrant activity. Arrhythmogenicity through such mechanisms have been discussed recently in reviews elsewhere (Hatem and Sanders, 2014; Venteclef et al., 2015; Hatem et al., 2016; Pandit et al., 2016; Samanta et al., 2016). Herein, we will focus on the characteristics of histo-pathology of adiposity in the myocardium in some of these diseases, (Christensen et al., 2008; Pouliopoulos et al., 2013; Haemers et al., 2015; Deshpande et al., 2016), especially in relation to (fibro) fatty infiltrates.

In obese humans and animal models, there is excess periatrial fat as well as fatty infiltration of the myocardium. Moreover, with obesity, myocardial cells have increased fat content, which is responsible for the attendant lipotoxic cardiomyopathy. It is hypothesized that a combination of factors, including other obesity-related comorbidities are responsible for generating substrates that favors arrhythmogenicity, and by mechanisms such as stated above. The role of excess myocardial fat, in the absence of other risk factors, has become of interest in a number of laboratories (Hatem et al., 2016; Pandit et al., 2016).

**Figure 3:**
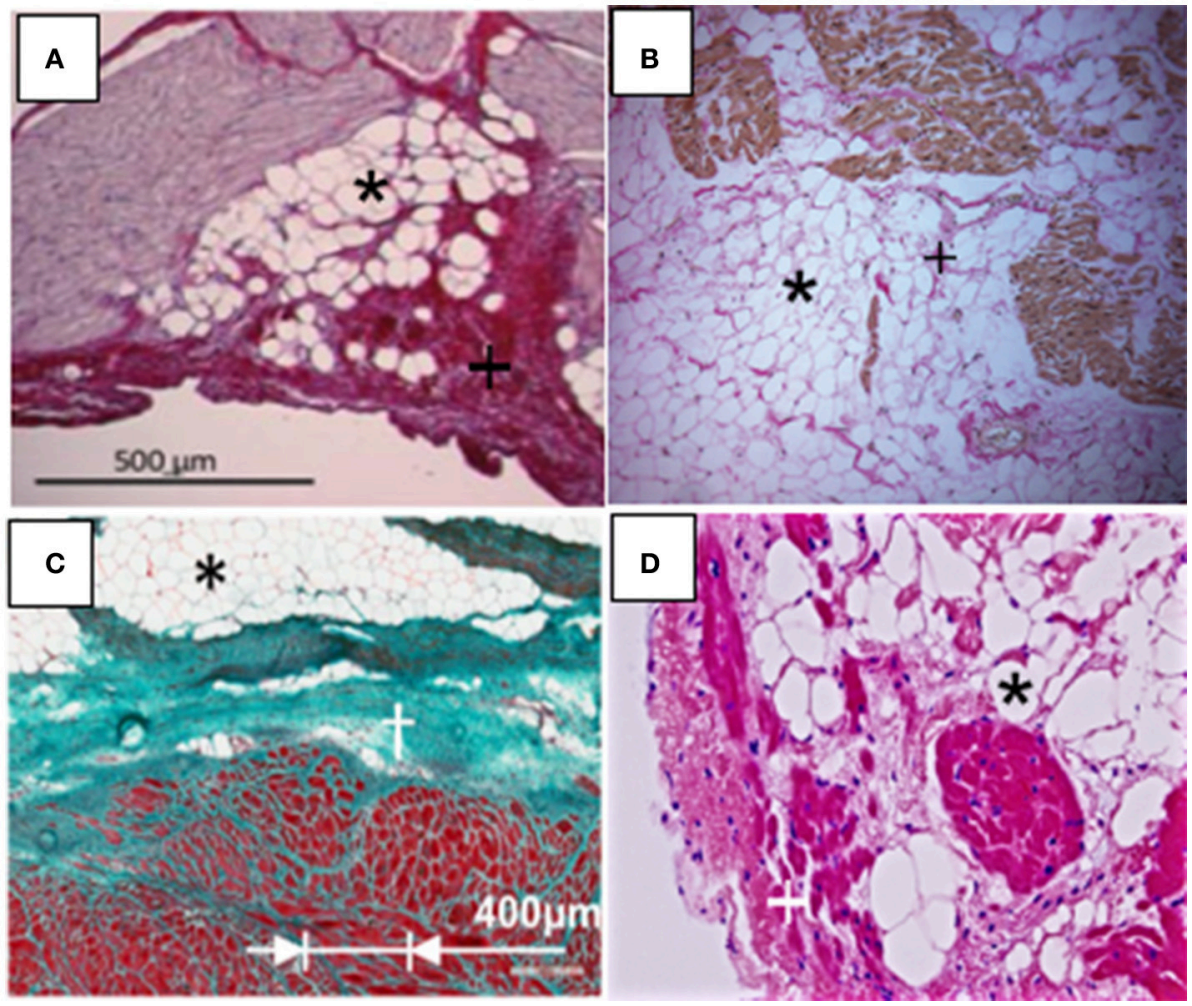
Disease associated myocardial fibro-fatty infiltrations (Fatty infiltrations, black asterisks; fibrosis, black or white crosses). **(A)** Atrial fibrillation (modified from Haemers et al., 2015). **(B)** Myotonic Dystrophy (magnification, 100X) (modified from Christensen et al., 2008). **(C)** Myocardial infarction (modified from Pouliopoulos et al., 2013). **(D)** Arrhythmogenic right ventricular cardiomyopathy (magnification, 200X). (Modified from Deshpande et al., 2016).

In addition to obesity, other diseases including AF, left ventricular hypertrophy, arrhythmogenic right ventricular cardiomyopathy (ARVC), post infarction cardiomyopathy, myotonic dystrophy, and Anderson-Fabry disease are associated with increased (fibro) fatty infiltration. Oftentimes in these diseases, excess adiposity is described as an increase in thickness (>5 mm) or fatty infiltration is reported on 0–3 scale, with the former representing highest extent of infiltration (Haemers et al., 2015; Mahajan et al., 2015). It should be noted that although some diseases show a constant fat–muscle ratio increase, during imaging, the extent of fatty infiltration might be difficult to determine. Thus, ischemia and hypertrophy, because of constancy of this ratio, have been reported to show a parallel increase in the mass of both tissue types (Gorter et al., 2008). Figure 3 shows characteristics of fatty and fibrous tissue infiltrates, exemplified in four different diseases. Thus, in normal weight humans, or in tachypaced ovine, extensive infiltrations have been reported with AF (Figure 3A; Haemers et al., 2015). Characteristically, during AF both left and right atrial myocardial walls have extensive fatty infiltration, patchy inflammation and interstitial fibrosis (Tanaka et al., 2007; Haemers et al., 2015). Previous CMRI based studies from patients with myotonic dystrophy have reported evidence of fatty infiltrations and fibrosis (Schmacht et al., 2016).

Figure 3B shows (fibro) fatty infiltration in a patient with cardiac myotonic dystrophy that mimics infiltrations such as seen in ARVC (Christensen et al., 2008). In this instance, histopathology following biopsies analyses shows fibrosis, fatty infiltration, and as well hypertrophied myocytes. Histological data of fibrofatty infiltration in Figure 3C were obtained from myocardial biopsy in the ovine model of chronic myocardial infarction (Pouliopoulos et al., 2013; Samanta et al., 2016). In the study, it was also reported that gap junctional [Connexin (Cx) 43] channels were significantly remodeled in myocytes within the scar zone, with Cx43 expression demonstrating extensive lateralization. Fatty infiltrations are well-characterized as a hallmark feature of ARVC, and are shown in Figure 3D (Deshpande et al., 2016). Additionally, there are abnormalities in expression of desmosomal proteins and hippo dependent signaling pathway, which ultimately compromise myocardial electrical coupling (Chen et al., 2014). From above studies, it is clear that there can be important implications of the direct physical contact between myocytes and adipocytes. For example, if the normal activity/cross talk between neighboring cells is altered, such as during metabolic derangement or with excessive electrical activity, there may be myocyte remodeling, potentially compromising myocardial function. Thus, the metabolic state of cardiac adipose tissue depot, when out of balance, such as with obesity and any attendant inflammation, should significantly contribute to myocardial structural and electrical remodeling. From a structural standpoint, fatty infiltrations may also create an anatomical block to the propagation of an electrical impulse. As previously noted, the anatomical block can create a substrate for fatal arrhythmias resulting from re-entry of the electrical signal.

It is generally recognized that arrhythmogenicity involves interactions of a trigger in combination with substrate (Iacobellis et al., 2005; Sacks and Fain, 2007; Cherian et al., 2012; Hatem and Sanders, 2014; Heijman et al., 2014; Pandit et al., 2016). On-going investigations are directed at understanding the role of excess adiposity, especially with respect to underlying signaling mechanisms, in the formation of such triggers and substrates. These studies address several important questions that remain unanswered. For example, what are the relative roles of different signaling pathways, such as by autocrine, paracrine and vasocrine pathways? (Sacks and Fain, 2007). There is also the question as to the source of the adipocytes/adipose tissue that infiltrate the myocardium in these disease conditions. Furthermore, what processes initiate myocardial infiltration and what are the molecular signaling events involved as the processes are initiated? (Chilukoti et al., 2015; Pandit et al., 2016). Understanding such processes and defining key molecular participants is necessary for proposing new therapeutic approaches to treat these diseases.

## Potential cellular and molecular mechanisms of adipose tissue-induced myocardial remodeling

As noted previously in a variety of reports (Iacobellis et al., 2005; Cherian et al., 2012; Hatem and Sanders, 2014; Pandit et al., 2016), adipocytes and other various cell types are present within the myocardium, constituting ~70% of total cardiac cell population. From a cellular standpoint, infiltration of the myocardium by abnormally proliferating cells can result in electrophysiological challenges reminiscent of microfibrosis (Wong et al., 2016). When infiltrates physically separate working myocardial cells, such challenges result in abnormalities in electrical propagation; including slowing of, and anisotropy in the conduction of the electrical impulse. Indeed, in a fairly recent large population study (Friedman et al., 2014; Wong et al., 2016), a relationship between pericardial fat and atrial conduction was demonstrated. Although not experimentally established, this association presumably reflects myocardial events mediated by above stated mechanisms.

**Figure 4:**
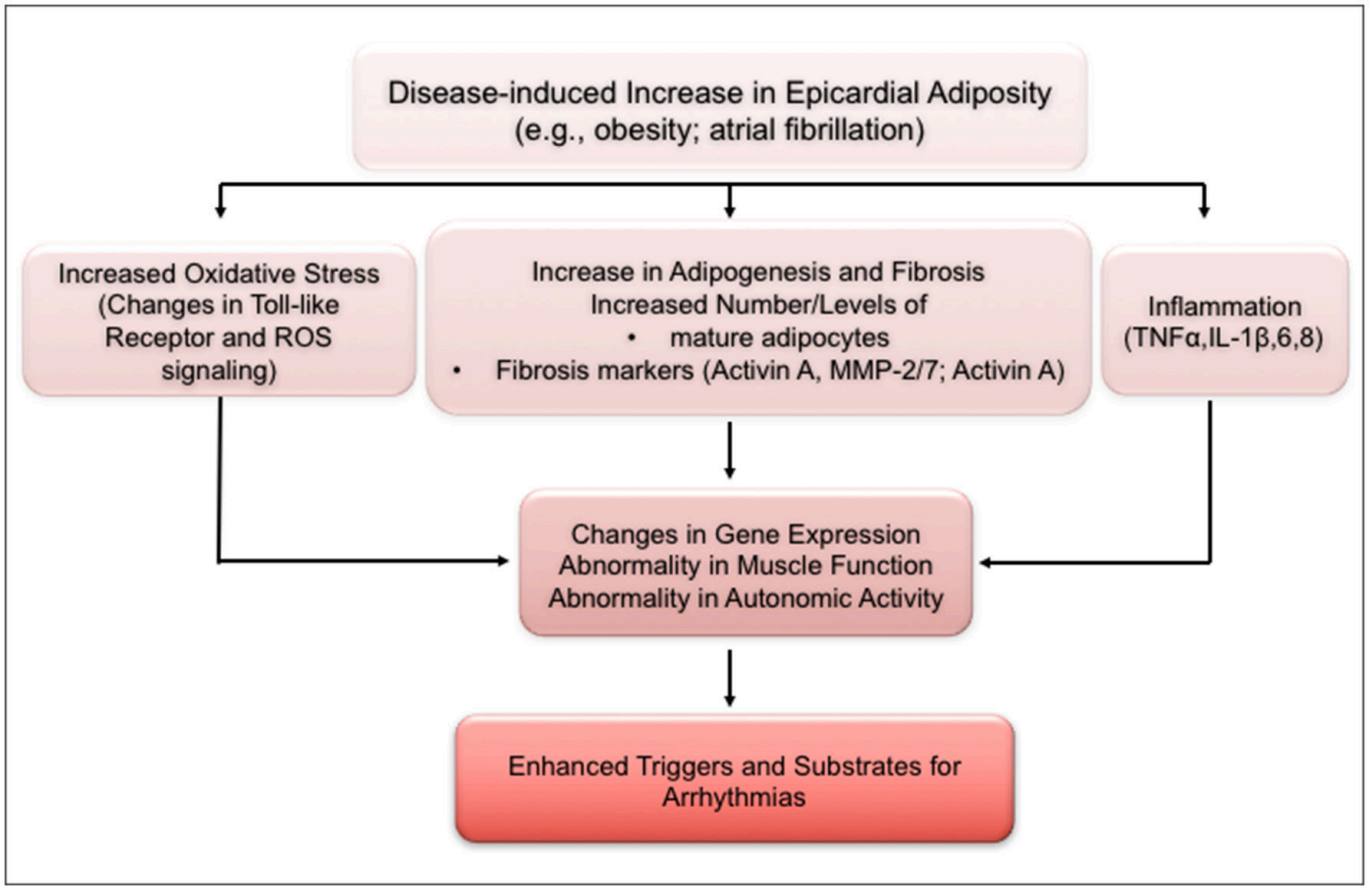
Schematic of mechanisms underlying increased arrhythmogenic risk with increased excess adiposity. ANS, autonomic nervous system; IL, interleukin; MMP, matrix metalloproteinase; ROS, reactive oxygen species; TLR, toll-like receptor; TNF, tumor necrosis factor. (Modified from Pandit et al., 2016).

Irrespective of depot site, each adipose tissue depot has an impressive metabolic activity, with a secretome that reflects the depot (Iacobellis et al., 2005; Hatem and Sanders, 2014; Venteclef et al., 2015). Biofactors released by cardiac adipose tissue include adipocytokines, e.g., adiponectin, resistin, fatty acid binding protein, leptin; angiogenic factors, including angiogenin, vascular endothelial growth factor; remodeling factors such as activin A; and a number of inflammatory cytokines, which include IL-1B, IL-6, TNF α, and monocyte chemotactic protein-1. These biofactors have been implicated in various heart diseases including coronary artery disease, obesity, and obesity-related coronary artery disease, heart failure, and diabetes. Depending on the biofactor, cardiac remodeling attendant to release of these agents are thought to be related to their effects, for example, on cell adhesion, extracellular matrix remodeling, dysregulation of intracellular calcium homeostasis, fibrosis, and inflammation (Hatem and Sanders, 2014). It is noteworthy that there are other cell types in the heart, and especially in myocardial adipose tissue, including pre-adipocytes, fibroblasts, lymphocytes, macrophages, and mast cells. Depending on (patho)-physiological conditions, these cells have been shown to release several of biofactors, including cytokines and free fatty acid, which are demonstrably capable of myocardial remodeling. Thus, we have shown that stearic acid, a major biofactor released by adipose tissue, causes extensive structural and electrical remodeling of sheep atrial myocytes (O'Connell et al., 2015). Cytokine production is linked to cardiomyocyte hypertrophy, and affects myocyte contractile activity (Martin and Blaxall, 2012). Moreover, properties of several ionic channels in rabbit atrial myocytes incubated in adipocyte media were significantly altered by the challenge (Lin et al., 2012). It must be emphasized, nevertheless, that with specific disease conditions, the biochemical properties of adipose tissue depots are qualitatively and quantitatively modified, an effect that will impact on the myocardial remodeling processes involved. The schematic in Figure 4 summarizes possible links and potential molecular mechanisms by which excess adiposity may lead to structural and electrical remodeling of the myocardium (Pandit et al., 2016). In the example provided, increase in epicardial fat, such as in obesity and/or atrial fibrillation, can lead to dysfunctionality in gene expression, muscle activity and in the autonomic nervous system, by mechanisms involving oxidative stress, fibro-fatty infiltration and inflammatory cytokines. As schematized, these processes are expected to enhance the generation of arrhythmogenic substrates and of triggering mechanisms for the formation of abnormal electrical impulses.

Overall, the subject matter of fatty infiltration has evolved from mere autopsy and myocardial biopsy reports to the extensive investigations of the electrophysiological consequences of such infiltrates. Indeed, advancements in imaging techniques are increasingly focused on improving our ability for more precise localization of epicardial fat depots and associated infiltrations. Such investigative endeavors will enhance our ability to determine correlations between such sites of infiltration and arrhythmogenic substrates. Another important aspect of excess adiposity and arrhythmogenicity is the origin and localization of cells involved in the process of fatty infiltration. Moreover, the types/subtypes of receptors, including the inflammation related toll-like receptors, which may be involved in the signaling of various biofactors, remain to be explored. Finally, given the evidence of pluripotency of some cells present in the myocardium, and especially in myocardial adipose tissues, understanding the variety of stimuli or factors that may initiate adipogenesis in the myocardium is also an important direction for future studies.

## Author contributions

JA: Developed the original idea for this mini review. He also wrote/edited the mini review. TH: Assisted in the writing/editing this review.

### Conflict of interest statement

The authors declare that the research was conducted in the absence of any commercial or financial relationships that could be construed as a potential conflict of interest.

## References

[B1] Bertaso A. G. Bertol D. Duncan B. B. Foppa M. (2013). Epicardial fat: definition, measurements and systematic review of main outcomes. Arq. Bras. Cardiol. 101, e18–e28. 10.5935/abc.20130138 23917514 PMC3998169

[B2] Burke S. Nagajyothi F. Thi M. M. Hanani M. Scherer P. E. Tanowitz H. B.. (2014). Adipocytes in both brown and white adipose tissue of adult mice are functionally connected via gap junctions: implications for Chagas disease. Microbes Infect. 16, 893–901. 10.1016/j.micinf.2014.08.006 25150689 PMC4353925

[B3] Chen S. N. Gurha P. Lombardi R. Ruggiero A. Willerson J. T. Marian A. J. (2014). The hippo pathway is activated and is a causal mechanism for adipogenesis in arrhythmogenic cardiomyopathy. Circ. Res. 114, 454–468. 10.1161/CIRCRESAHA.114.302810 24276085 PMC3946717

[B4] Cherian S. Lopaschuk G. D. Carvalho E. (2012). Cellular cross-talk between epicardial adipose tissue and myocardium in relation to the pathogenesis of cardiovascular disease. Am. J. Physiol. Endocrinol. Metab. 303, E937–E949. 10.1152/ajpendo.00061.2012 22895783

[B5] Chilukoti R. K. Giese A. Malenke W. Homuth G. Bukowska A. Goette A.. (2015). Atrial fibrillation and rapid acute pacing regulate adipocyte/adipositas-related gene expression in the atria. Int. J. Cardiol. 187, 604–613. 10.1016/j.ijcard.2015.03.072 25863735

[B6] Christensen A. H. Bundgaard H. Schwartz M. Hansen S. H. Svendsen J. H. (2008). Cardiac myotonic dystrophy mimicking arrhythmogenic right ventricular cardiomyopathy in a young sudden cardiac death victim. Circ. Arrhythm. Electrophysiol. 1, 317–320. 10.1161/CIRCEP.108.785865 19808424

[B7] Corradi D. Maestri R. Callegari S. Pastori P. Goldoni M. Luong T. V.. (2004). The ventricular epicardial fat is related to the myocardial mass in normal, ischemic and hypertrophic hearts. Cardiovasc. Pathol. 13, 313–316. 10.1016/j.carpath.2004.08.005 15556777

[B8] Deshpande S. R. Herman H. K. Quigley P. C. Shinnick J. K. Cundiff C. A. Caltharp S.. (2016). Arrhythmogenic Right Ventricular Cardiomyopathy/Dysplasia (ARVC/D): review of 16 pediatric cases and a proposal of modified pediatric criteria. Pediatr. Cardiol. 37, 646–655. 10.1007/s00246-015-1327-x 26743400

[B9] Friedman D. J. Wang N. Meigs J. B. Hoffmann U. Massaro J. M. Fox C. S.. (2014). Pericardial fat is associated with atrial conduction: the framingham heart study. J. Am. Heart. Assoc. 3:e000477. 10.1161/JAHA.113.000477 24595189 PMC4187474

[B10] Gorter P. M. van Lindert A. S. de Vos A. M. Meijs M. F. van der Graaf Y. Doevendans P. A.. (2008). Quantification of epicardial and peri-coronary fat using cardiac computed tomography; reproducibility and relation with obesity and metabolic syndrome in patients suspected of coronary artery disease. Atherosclerosis 197, 896–903. 10.1016/j.atherosclerosis.2007.08.016 17884060

[B11] Haemers P. Hamdi H. Guedj K. Suffee N. Farahmand P. Popovic N.. (2015). Atrial fibrillation is associated with the fibrotic remodelling of adipose tissue in the subepicardium of human and sheep atria. Eur. Heart J. 38, 53–61. 10.1093/eurheartj/ehv625 26612579

[B12] Hatem S. N. Redheuil A. Gandjbakhch E. (2016). Cardiac adipose tissue and atrial fibrillation: the perils of adiposity. Cardiovasc. Res. 109, 502–509. 10.1093/cvr/cvw001 26790475

[B13] Hatem S. N. Sanders P. (2014). Epicardial adipose tissue and atrial fibrillation. Cardiovasc. Res. 102, 205–213. 10.1093/cvr/cvu045 24648445

[B14] Heijman J. Voigt N. Nattel S. Dobrev D. (2014). Cellular and molecular electrophysiology of atrial fibrillation initiation, maintenance, and progression. Circ. Res. 114, 1483–1499. 10.1161/CIRCRESAHA.114.302226 24763466

[B15] Iacobellis G. Corradi D. Sharma A. M. (2005). Epicardial adipose tissue: anatomic, biomolecular and clinical relationships with the heart. Nat. Clin. Pract. Cardiovasc. Med. 2, 536–543. 10.1038/ncpcardio0319 16186852

[B16] Iozzo P. (2011). Myocardial, perivascular, and epicardial fat. Diabetes Care 34(Suppl. 2), S371–S379. 10.2337/dc11-s250 21525485 PMC3632210

[B17] Lin Y. K. Chen Y. C. Chen J. H. Chen S. A. Chen Y. J. (2012). Adipocytes modulate the electrophysiology of atrial myocytes: implications in obesity-induced atrial fibrillation. Basic Res. Cardiol. 107:293. 10.1007/s00395-012-0293-1 22886089

[B18] Mahajan R. Lau D. H. Brooks A. G. Shipp N. J. Manavis J. Wood J. P.. (2015). Electrophysiological, electroanatomical, and structural remodeling of the atria as consequences of sustained obesity. J. Am. Coll. Cardiol. 66, 1–11. 10.1016/j.jacc.2015.04.058 26139051

[B19] Marchington J. M. Mattacks C. A. Pond C. M. (1989). Adipose tissue in the mammalian heart and pericardium: Structure, foetal development and biochemical properties. Comp. Biochem. Physiol. B 94, 225–232. 10.1016/0305-0491(89)90337-4 2591189

[B20] Martin M. L. Blaxall B. C. (2012). Cardiac intercellular communication: are myocytes and fibroblasts fair-weather friends? J. Cardiovasc. Transl. Res. 5, 768–782. 10.1007/s12265-012-9404-5 23015462 PMC3518575

[B21] O'Connell R. P. Musa H. Gomez M. S. Avula U. M. Herron T. J. Kalifa J.. (2015). Free fatty acid effects on the atrial myocardium: membrane ionic currents are remodeled by the disruption of T-tubular architecture. PLoS ONE. 10:e0133052. 10.1371/journal.pone.0133052 26274906 PMC4537212

[B22] Pandit S. V. Anumonwo J. Jalife J. (2016). Atrial fibrillation susceptibility in obesity: an excess adiposity and fibrosis complicity? Circ. Res. 118, 1468–1471. 10.1161/CIRCRESAHA.116.308686 27174946 PMC4869997

[B23] Pouliopoulos J. Chik W. W. Kanthan A. Sivagangabalan G. Barry M. A. Fahmy P. N.. (2013). Intramyocardial adiposity after myocardial infarction: new implications of a substrate for ventricular tachycardia. Circulation 128, 2296–2308. 10.1161/CIRCULATIONAHA.113.002238 24036606

[B24] Sacks H. S. Fain J. N. (2007). Human epicardial adipose tissue: a review. Am. Heart J. 153, 907–917. 10.1016/j.ahj.2007.03.019 17540190

[B25] Samanta R. Pouliopoulos J. Thiagalingam A. Kovoor P. (2016). Role of adipose tissue in the pathogenesis of cardiac arrhythmias. Heart Rhythm. 13, 311–320. 10.1016/j.hrthm.2015.08.016 26277495

[B26] Schmacht L. Traber J. Grieben U. Utz W. Dieringer M. A. Kellman P.. (2016). Cardiac involvement in myotonic dystrophy type 2 patients with preserved ejection fraction: detection by cardiovascular magnetic resonance. Circ. Cardiovasc. Imaging 9:e004615 10.1161/CIRCIMAGING.115.004615 27363857

[B27] Sen-Chowdhry S. Morgan R. D. Chambers J. C. McKenna W. J. (2010). Arrhythmogenic cardiomyopathy: etiology, diagnosis, and treatment. Annu. Rev. Med. 61, 233–253. 10.1146/annurev.med.052208.130419 20059337

[B28] Tanaka K. Zlochiver S. Vikstrom K. L. Yamazaki M. Moreno J. Klos M.. (2007). Spatial distribution of fibrosis governs fibrillation wave dynamics in the posterior left atrium during heart failure. Circ. Res. 101, 839–847. 10.1161/CIRCRESAHA.107.153858 17704207

[B29] Thanassoulis G. Massaro J. M. O'Donnell C. J. Hoffmann U. Levy D. Ellinor P. T.. (2010). Pericardial fat is associated with prevalent atrial fibrillation: the Framingham Heart Study. Circ. Arrhythm. Electrophysiol. 3, 345–350. 10.1161/CIRCEP.109.912055 20558845 PMC2953855

[B30] Venteclef N. Guglielmi V. Balse E. Gaborit B. Cotillard A. Atassi F.. (2015). Human epicardial adipose tissue induces fibrosis of the atrial myocardium through the secretion of adipo-fibrokines. Eur. Heart J. 36, 795a–805a. 10.1093/eurheartj/eht099 23525094

[B31] Wong C. X. Ganesan A. N. Selvanayagam J. B. (2016). Epicardial fat and atrial fibrillation: current evidence, potential mechanisms, clinical implications, and future directions. Eur. Heart J. 38, 1294–1302. 10.1093/eurheartj/ehw045 26935271

